# Sleep in the Postpartum: Characteristics of First-Time, Healthy Mothers

**DOI:** 10.1155/2017/8520358

**Published:** 2017-10-17

**Authors:** Laura Creti, Eva Libman, Dorrie Rizzo, Catherine S. Fichten, Sally Bailes, Dieu-Ly Tran, Phyllis Zelkowitz

**Affiliations:** ^1^Jewish General Hospital, Montreal, QC, Canada; ^2^McGill University, Montreal, QC, Canada; ^3^Université de Montréal, Montreal, QC, Canada; ^4^Dawson College, Montreal, QC, Canada

## Abstract

Goals for the present study were to (a) describe the sleep of healthy new mothers over a 6-month postpartum period, (b) examine how sleep quality relates to daytime levels of fatigue and sleepiness, and (c) evaluate the relationship between mothers' and infants' sleep parameters. The sample consisted of 37 healthy, partnered, first-time mothers who had experienced full-term vaginal birth and had a healthy infant. We investigated infants' sleep parameters and mothers' sleep, mood, and daytime functioning 2 and 6 months postpartum. We found that at 2 months postpartum, mothers reported sleeping 6 hours at night and just under one hour during the day. Despite relatively frequent nocturnal awakenings, mothers experienced minimal insomnia, nonrefreshing sleep, anxiety, depression, daytime sleepiness, or fatigue at either 2 or 6 months. The most robust relationship between mothers' and infants' sleep was in the number of nocturnal sleep-wake episodes. Of note is that none of the infant sleep parameters was related to mothers' anxiety, depression, fatigue, sleepiness, or nonrefreshing sleep at either time period. Our results indicate that (1) selected low risk new mothers are resilient in terms of sleep quality, daytime functioning, and mood and (2) these are independent of their infants' sleep parameters.

## 1. Introduction

Although there is a long history of investigation into the implications of sleep disruption and sleep deprivation in vulnerable populations (e.g., medical residents, shift workers, and commercial drivers [1–3]), little is known of how sleep disruption affects new mothers' performance. As maternal sleep quality in the perinatal period became of increasing interest, a seminal study by Signal et al. [4] documented that the greatest disruption occurred at one week postpartum. Another study examined how the manifestation of daytime sleepiness related to sleep disturbance in postpartum women [5]. These researchers noted that over 50% of women were still experiencing excessive daytime sleepiness at week 18.

While it seems evident that soon after birth, the mother's sleep is greatly affected by many things, such as nighttime feedings and other challenges of infant care, little has been researched about the evolution of mother-infant sleep (both nighttime and daytime) and the progression from the early postpartum period to the later months, as infants' sleep and metabolic circadian rhythms develop. The present study examines change in mother-infant sleep at six months as compared with two months postpartum.

It is well known that postpartum major depression is a serious illness, occurring in approximately 13% of mothers, that negatively affects both mother and child psychological adjustment in the long term [6]. Meta-analyses by Beck and Perkins [7] and by O'Hara and Swain [6] examined multiple factors predicting postpartum depression. However, neither investigation reported any sleep-related variables. Similarly, Martini et al. [8] did not include sleep variables when examining depression and anxiety in peripartum women, although we can assume that poor sleep may have been confounded in the measurement of depression.

The interaction of maternal sleep and mood has received increasing interest in the literature. For example, Lawson et al. [9], in a systematic review, found a moderately strong relationship between disrupted and poor sleep and postpartum depression. Other studies have examined the association of maternal sleep disturbance with other postnatal aspects such as postnatal maternal attachment [10], impaired maternal-infant bonding [11]. Tomfohr et al. [12] evaluated sleep quality and mood at three time periods during pregnancy and at three months postpartum. They concluded that the groups with the worst sleep quality during pregnancy were the most likely to experience symptoms of depression postpartum.

It appears to be well established that during the perinatal period, there is an association between poor sleep quality and impaired daytime and psychological functioning. It is not yet clear whether improvement in sleep disruption over time is associated with change in depressive aspects. We know little about the potential impact of prolonged, extrinsically induced sleep disruption on subsequent development of insomnia in general. There is even less information for postpartum women specifically, where sleep disruption, particularly in the early weeks, is prevalent. By deliberately selecting a low risk sample of women, the present study focuses on the universal experience of sleep disruption postpartum and limits as much as possible some of the known risk factors (single mother, low socioeconomic status, infant morbidity, etc.).

## 2. Present Investigation

In the present study, we investigated the association between sleep quality, depression, anxiety, and daytime variables (sleepiness and fatigue). We selected a sample of healthy, first-time new mothers who were living with a partner and had a healthy infant after having experienced full-term vaginal birth. This relatively problem-free sample was selected to appreciate more fully the potential interplay of sleep disruption, insomnia, and postpartum psychological adjustment. We anticipated that, in our preselected healthy sample, we would not find a high frequency of anxiety and depression. Our aim was to investigate whether sleep disruption itself would increase frequency or intensity of emotional disturbance.

The specific aims of the study were to describe the sleep of healthy new mothers over a 6-month postpartum period and to examine how sleep quality relates to their daytime levels of fatigue and sleepiness. We also evaluated the relationship between mothers' and infants' sleep parameters: how mothers' mild to moderate levels of insomnia in the postpartum might relate to maternal depression and anxiety and how all these variables evolve over time. Specifically, we wondered (a) whether the pronounced sleep disruption experienced at two months postpartum, with associated higher levels of daytime fatigue and sleepiness, would precipitate increased insomnia, anxiety, and depressive symptoms and (b) what might happen to such symptoms at six months postpartum, when infant sleep is generally more consolidated and sleep disruption related to infant care is reduced.

## 3. Materials and Methods

### 3.1. Participants

Participants were 37 first-time mothers (mean age = 29.22, SD = 5.51). They were recruited from the postpartum unit of a large urban general hospital. Only mothers living with their partners, who had a healthy, normal birth weight infant following an uncomplicated vaginal birth, were selected for this study.

#### 3.1.1. Sample Characteristics

Twenty-four of the women were married while 13 were living with a partner. The average number of years of cohabitation for the entire sample was 4.52 (SD = 3.33) years with only 4 couples having lived together for less than one year upon entering the study. Only one mother worked outside the home at two months postpartum; five were working at six months postpartum. The average number of years of mother's education was 16.14 (SD = 3.18), with the majority of women (83.8%) having completed postsecondary education. The sample's ethnic background was diverse, with the majority (56.8%) of mothers having been born outside of Canada. The average number of years they lived in Canada was 16.75 (SD = 13.64).

68% of the infants were male. Infants' mean gestational age was 39.28 weeks (SD = 1.75). 84% of infants were breastfed a two months; 50% were still being breastfed at 6 months. 78% of infants were sleeping in the same room as the mother at two months; 41% of infants were still in mother's bedroom at six months.

### 3.2. Measures

#### 3.2.1. Sleep Symptom Checklist (SSC) [13]

The SSC is a 21-item survey of a broad range of symptoms that are both directly and indirectly related to sleep disorders. Respondents rate each symptom, including poor sleep onset, poor sleep maintenance, waking too early, nonrefreshing sleep, and poor sleep quality, for its severity from 0 (not at all) to 3 (very severe) based on the previous month. Temporal stability of the SSC is acceptable (total score *r* = 0.79, *p* < .01; Cronbach's alpha = 0.74).

#### 3.2.2. Insomnia Severity Index (ISI) [14]

This frequently used seven-item measure has been used to detect insomnia. Psychometric evaluation shows that the measure has excellent internal consistency (Cronbach's alpha ranges from .90 to .91) and that scores reflect pre-to posttherapy changes. Scores less than or equal to 7 indicate no insomnia, scores between 8 and 14 indicate subthreshold insomnia, scores between 15 and 21 indicate the presence of a moderate level of insomnia, and scores between 22 and 28 indicate a severe insomnia in community samples.

#### 3.2.3. Sleep Diary

A 7-day sleep diary was used to evaluate sleep of new mothers and their infants. Since they were expected to have several sleep episodes throughout the day and night, mothers were asked to record when they actually slept over a 24-hour period. They were also asked to record when their infants slept. Three scores are derived from this measure. Total 24-hour sleep time was calculated by adding the duration of sleep at all episodes; total nighttime sleep was the total time slept between 9:00 PM and 9:00 AM, while total daytime sleep was the total amount of time slept between 9:00 AM and 9:00 PM [15].

#### 3.2.4. Empirical Sleepiness and Fatigue Scales [16]

Since existing scales measuring fatigue and sleepiness confounded the two concepts, the Empirical Sleepiness and Fatigue Scales were developed through correlation and factor analysis of all items from four popular measures purporting to measure sleepiness and fatigue. The two Empirical Scales represent different constructs with distinctive patterns of associations and were only minimally correlated with each other. The Empirical Sleepiness Scale consists of 6 items from the Epworth Sleepiness Scale and the Empirical Fatigue Scale consists of 1 item from the Fatigue Severity Scale [17], and 2 from the Chalder Fatigue Scale [18]. Sleepiness is generally defined as sleep propensity and fatigue as diminished energy. The measure has good internal consistency and test-retest reliability. Higher scores indicate greater sleepiness or fatigue.

#### 3.2.5. Edinburgh Postnatal Depression Scale (EPDS) [19]

This is one of the most widely used measures of perinatal depressive symptomatology [20]. The 10 items ask respondents to report on their feelings during the past 7 days and the scale only requires 5 minutes to complete. A score of 13 or higher has optimal sensitivity and specificity in relationship to a diagnosis of major depression.

#### 3.2.6. Generalized Anxiety Disorder (GAD-7 Scale) [21]

This 7-item questionnaire asks about symptoms of anxiety in the past two weeks. Symptoms are rated on a 4-point scale, ranging from “not at all” to “nearly every day.” The measure has good internal and test-retest reliability and has been shown to be valid in relation to a diagnosis of generalized anxiety disorder made by clinical interview. It has been shown to be an effective screening tool for anxiety disorders. Scores of 5, 10, and 15 are taken as the cutoff points for mild, moderate, and severe anxiety, respectively. When used as a screening tool, further evaluation is recommended when the score is 10 or greater.

### 3.3. Procedure

The present study is part of a larger investigation of mental health and illness in pregnant and perinatal women (e.g., [22, 23]). In the present investigation, we focused on new mothers' sleep-related perceptions and experiences postpartum: a transient period of interrupted and shortened sleep caused by their caring for their newborn infants. Data collection took place in consenting participants' homes, at 2 and at 6 months postpartum. Mothers completed the battery of questionnaires as well as the 7-day Sleep Diaries for themselves and their infants.

## 4. Results

### 4.1. Sample Characteristics

24 of the women were married while 13 were living with a partner. The average number of years of cohabitation for the entire sample was 4.52 (SD = 3.33) years with only 4 couples having lived together for less than 1 year upon entering the study. Only one mother worked outside the home at 2 months postpartum and 5 were working at 6 months postpartum. The average number of years of mother's education was 16.14 (SD = 3.18), with the majority of women (83.8%) having completed postsecondary education. The sample's ethnic background was diverse, with the majority (56.8%) of mothers having been born outside of Canada. The average number of years they lived in Canada was 16.75 (SD = 13.64).

### 4.2. Mothers' and Infants' Sleep

Based on ISI scores, when mothers who had no insomnia were compared with those who had some insomnia at 6 months postpartum, there were no significant differences found on infant sex, *X*^2^(1, *N* = 37) = 1.097, *p* = .295, infants who were breastfed at night, *X*^2^(1, *N* = 37) = 1.47, *p* = .23, or where infants slept, *X*^2^(1, *N* = 37) = 2.10, *p* = .147.

#### 4.2.1. Total Sleep Time and Sleep Episodes

As seen in Table 1, at 2 months, mother's average nighttime total sleep time (TST) was 6.29 hours, with a mean of 2-1/2 sleep episodes. At night, infants slept an average of 7.76 hours, with an average of 3 sleep episodes. During the day infants slept an average 4-1/2 hours, in 3-1/2 discrete sleep episodes, while mothers slept less than one hour. At 6 months, both mothers and infants' nocturnal TST increased significantly and the number of sleep episodes decreased significantly. The reverse significance pattern is evident for daytime sleep.

#### 4.2.2. Insomnia Severity

Table 1 also shows that mean ISI scores were in the “subthreshold insomnia” range at both 2 and 6 months postpartum. Frequencies in Table 2 provide information on insomnia frequencies at both 2 and 6 months. None of the mothers scored in the severe insomnia category at either time.

With respect to difficulty initiating or maintaining sleep (DIMS), means in Table 1 show that mothers had little difficulty initiating or maintaining sleep or waking too early at both time periods. The number of participants who endorsed none or mild symptoms (a rating of 0 or 1) versus moderate or severe symptoms (a rating of 2 or 3) on the SSC was also examined (see Table 2). In summary, although most mothers (from 68% to 89% of the sample) reported no or mild DIMS, 5% to 16% reported experiencing moderate to severe DIMS at both time periods.

### 4.3. Daytime Functioning

#### 4.3.1. Sleepiness and Fatigue

Table 1 shows that there was a significant decrease in mean sleepiness from 2 to 6 months but no significant change in fatigue. Table 2 provides frequencies.

#### 4.3.2. Nonrefreshing Sleep

Means for nonrefreshing sleep were low and did not change significantly over time (Table 1). Table 2 shows that only 8 mothers reported experiencing moderate or severe nonrefreshing sleep at 2 months and only 6 mothers at 6 months.

#### 4.3.3. Depression and Anxiety Postpartum

Table 1 shows that average depression (EPDS) scores were in the normal range and did not change significantly over time.  Table 3 shows that few mothers scored in the possible clinical range at both times. Even so, scores decreased significantly at 6 months postpartum (see Table 1). Although the number of mothers scoring in the clinical range was similar for depression and anxiety, again, very few mothers scored in the clinical range on both variables at 6 months.

### 4.4. Evolution of Mothers' Sleep and Psychological Adjustment over Time

In a series of analyses, we tracked the trajectory of change from 2 to 6 months in individual women. Tables 2 and 3 provide group means and the section below provides information on trajectories.

#### 4.4.1. Insomnia Severity

To examine the evolution of insomnia over time, the sample was divided into two groups: below ISI subthreshold score of 8 and equal to or above 8. The data indicate that 73% of mothers who scored below the threshold score at 2 months remained below the threshold at 6 months; 59% who scored above the threshold at 2 months remained above the threshold. Twenty-four percent of mothers improved and 11% deteriorated.

#### 4.4.2. DIMS

The evolution of problems of initiating and maintaining sleep was examined by separating individuals into high (ratings of 2 or 3) and low severity (ratings of 0 or 1) on SSC variables at 2 months. Sleep onset problems remained low at 6 months for 84% of mothers, while 5% of mothers deteriorated, 5% improved, and 5% remained high. For sleep maintenance problems at 6 months, 70% remained low, 19% improved, 5% deteriorated, and 5% remained high. For early morning awakening problems, at 6 months 46% remained low, 32% improved, 5% deteriorated, and 16% remained high.

#### 4.4.3. Daytime Functioning

The evolution of sleepiness and fatigue was examined by separating individuals above and below each Scale's means at 2 months.

For sleepiness, 46% remained below the mean, 32% improved, 5% deteriorated, and 17% remained above the mean. For fatigue, 54% remained below the mean, 16% improved, 8% deteriorated, and 22% remained above the mean.

The evolution of problems of nonrefreshing sleep was examined by separating individuals into high and low severity on the SSC item at 2 months. At 6 months 73% of mothers remained low, 11% improved, 5% deteriorated, and 11% remained high.

#### 4.4.4. Depression and Anxiety

The evolution of depression indicates that over time 31 (84%) mothers remained below the cutoff score, 3 (8%) improved to below the cutoff, 2 (5%) deteriorated (changed from below the cutoff score at 2 months to above at 6 months), and 1 (3%) remained above the cutoff score. The evolution of anxiety over time indicates that 31 (84%) of mothers remained below the cutoff score of 10, 3 (8%) improved, 2 (5%) deteriorated, and 1 (3%) remained above the cutoff score.

### 4.5. Relationships Among Variables

#### 4.5.1. How Is the Sleep of New Mothers Related to Daytime Variables?

Correlations in Table 4 indicate that only one of the nocturnal sleep variables, Insomnia Severity Scale (ISI), was correlated significantly with all three daytime variables at both 2 and 6 months postpartum. At 6 months sleepiness scores were also correlated with Sleep Onset and Sleep Maintenance Problems and nonrefreshing sleep was significantly correlated with Sleep Onset Problems, Sleep Maintenance Problems, Early Morning Awakening, and lower Nighttime TST.

#### 4.5.2. Links between Depression and Anxiety at 2 and 6 Months on the One Hand and Mothers' Daytime and Nocturnal Scores on the Other

Table 5 shows that, at 2 months, despite scores being in the generally normal range, both depression and anxiety were significantly correlated with nocturnal sleep and daytime sleep-related variables. Depression was also correlated with sleep onset problem scores. At 6 months, both depression and anxiety were correlated with Fatigue and Sleepiness Scale scores, and lower maternal nocturnal total sleep time. Anxiety was also significantly correlated with poor sleep. There were no significant correlations between maternal psychological adjustment and infant sleep characteristics.

#### 4.5.3. How Are Mothers' Sleep and Daytime Adjustment Related to Their Infants' Sleep over Time?

Infants' sleep variables were significantly correlated with more of the mothers' sleep variables at 2 months compared to 6 months (Table 6). The most consistent highly significant correlation over time was the positive relationship between mothers' and infants' nighttime sleep episodes. Only one other correlation was significant at 6 months postpartum: mothers' daytime sleepiness was related to shorter infant daytime TST; depression and anxiety were unrelated to infant sleep characteristics at both time periods. 

## 5. Discussion

### 5.1. Sleep and Daytime Characteristics

At 2 months postpartum, the healthy new mothers in our sample reported sleeping about 6 hours at night and just under an hour during the day. Although seemingly mothers did not take full advantage of their infants' daytime sleep to nap themselves, the fact that infants, who slept about 4-1/2 hours during the day, did so in an average of three separate episodes may account for this.

Our study confirmed that, at two months postpartum, mothers' and infants' nocturnal total sleep times were closely related, and the number of mothers' nocturnal awakenings corresponded closely to reported infants' awakenings. As expected, both mothers and infants slept more hours with fewer interruptions at 6 months as compared with two months postpartum.

When examining daytime sleepiness, contrary to the findings of Filtness et al. [5], who found that over 50% of the women in their sample were still experiencing excessive daytime sleepiness at week 18, we found that there was a substantial and significant improvement at 6 months and that 80% of mothers were in the low sleepiness group.

Across variables, most mothers tended to remain unchanged, mirroring the recently noted stability in infant sleep behaviours [24]. They experienced low insomnia severity and daytime and psychological symptoms at 6 months. Changes tended to be towards improvement rather than deterioration, both when we examined mean scores as well as when we examined trajectories by tracing what happened to individual women. This suggests that, in this generally healthy sample, most mothers are quite resilient in the context of the temporary disruption of a needy infant.

It is important to note, however, that when we examined insomnia-related parameters over time, a small proportion of mothers failed to improve. Similarly, with respect to daytime trajectories, a small number of mothers remained sleepy and fatigued at six months. A similar small percentage continued to experience nonrefreshing sleep, and slightly elevated anxiety and depression. Correlational data indicated that nonrefreshing sleep, an important marker of sleep quality [25], was significantly related to virtually all nocturnal sleep variables. This pattern of findings may reflect a preexisting tendency to sleep difficulty, anxiety, and impaired mood in a small percentage of mothers that does not respond to what is usually considered as the improved conditions of the 6-month postpartum period. This interpretation would appear to be supported by the reported role of personality [26] and genetics [27] in maternal insomnia.

Tomfohr et al. [12] reported that the groups with the worst sleep quality during pregnancy were the most likely to experience symptoms of depression postpartum. In our sample of women, all of whom were postpartum, we found a significant link between depression and poor sleep quality only at 2 months. However, we found that there were significant correlations between depression scores and less total sleep time, a variable that was also related to anxiety.

It is especially noteworthy that no infant sleep parameters were related to mothers' anxiety or depression. Moreover, at 2 months there was no significant relationship between the mothers' fatigue, sleepiness, and nonrefreshing sleep with infants' sleep parameters. Indeed, even at 6 months, only two of the possible 48 coefficients were significant, suggesting that infants' sleep was essentially unrelated to mothers' daytime functioning.

### 5.2. Limitations and Future Research

Our findings constitute a snapshot of two one-week self-reported postpartum periods, at two months and six months. No objective sleep data are reported. In addition, from the point of view of interpreting sleep problems and variations in anxiety and depression in these new mothers, it would have been useful to have examined prior habitual sleep and psychological adjustment.

One might hypothesize that the elevated scores in insomnia-related variables for a small segment of our sample reflect an intrinsic insomnia risk factor, which is triggered by the stresses of first-time motherhood. This hypothesis is supported by a recent qualitative study which evaluated sleep quality in a high risk for poor postpartum sleep sample, low income African American mothers [28]. To evaluate this hypothesis, one would need to carry out a prospective study, where a large sample of women are evaluated on sleep patterns, daytime functioning, and psychological functioning beginning at the prepregnancy period and followed during pregnancy and postpartum. The model of Tomfohr and colleagues [12], which identified four trajectory patterns in a heterogeneous sample of women across the perinatal period, could provide an interesting structure for longer term follow-up, both before, during, and after the postpartum period.

## 6. Conclusions

Two findings are highlighted in the present study:

1. We assumed that we had recruited a low risk sample and, in fact, in terms of sleep quality, daytime functioning, anxiety, and depression, these new mothers did manifest an impressive resilience to the disruptive aspects of the postpartum period. This is contrary to what was found in Zambrano et al. [28] high risk sample, where sleep quality was poor, and anxiety and depression were high.
2. Notably, in both our low risk and Zambrano et al.'s [28] high risk samples of postpartum women, sleep quality, daytime functioning, and mood were independent of their infants' sleep parameters. These results suggest that both sleep-related resilience and psychological adjustment are intrinsic to the mother and are not dependent on their infants' sleep pattern.

## Figures and Tables

**Table 1:** Comparisons of 2 and 6 months' nighttime and daytime variables.

Variables	2 months		6 months		*t*	df	Sig.
	Mean	SD	Mean	SD			
Nighttime variables							
Poor sleep quality (SSC)^1^	0.86	0.92	0.84	*0.96*	0.16	36	.872
Insomnia Severity Scale (ISI)^2^	9.14	*4.89*	7.51	*5.42*	1.79	36	.081
Difficulty initiating or maintaining sleep (DIMS)							
Sleep onset problem (SSC)^1^	0.43	*0.69*	0.51	*0.77*	−0.65	36	.520
Sleep maintenance problem (SSC)^1^	0.65	*0.92*	0.65	*0.82*	0.00	36	1.000
Waking too early (SSC)^1^	1.11	*1.49*	1.11	*1.26*	0.00	35	1.000
Nighttime TST, mother (diary)	6.29	*1.15*	7.22	*0.80*	−4.03	35	.000
Nighttime TST, infant (diary)	7.76	*1.07*	9.14	*1.01*	−6.45	35	.000
Number of nighttime sleep episodes, mother (diary)	2.44	*0.72*	2.02	*0.95*	2.83	35	.008
Number of nighttime sleep episodes, infant (diary)	3.20	*0.98*	2.41	*1.24*	3.84	35	.000
Daytime variables							
Fatigue Scale (ESFS)^3^	9.05	*4.85*	8.35	*4.50*	0.96	36	.345
Sleepiness Scale (ESFS)^4^	6.32	*4.18*	4.65	*4.07*	2.81	36	.008
Nonrefreshing sleep (SSC)^1^	0.81	*0.91*	0.73	*0.99*	0.55	36	.584
Sleep							
Daytime TST, mother (diary)	0.93	*0.68*	0.50	*0.54*	4.10	35	.000
Daytime TST, infant (diary)	4.53	*1.21*	3.05	*0.91*	8.11	35	.000
Number of daytime sleep episodes, mother (diary)	0.74	*0.42*	0.47	*0.44*	3.95	35	.000
Number of daytime sleep episodes, infant (diary)	3.42	*1.00*	2.79	*0.84*	5.90	35	.000
Mood							
Anxiety (GAD-7)^5^	4.78	*3.43*	3.22	*3.18*	2.68	36	.011
Depression (EPDS)^6^	7.11	*3.85*	5.95	*3.27*	1.88	36	.068

^1^SSC: Sleep Symptom Checklist. All variables: 0–3 Likert scale: the lower the value the better the functioning; ^2^ISI: Insomnia Severity Index, Scores >= 15 indicate moderate severity; ^3^ESFS: Empirical Fatigue Scale. The lower the scores the better the functioning. Range = 0–19; ^4^ESFS: Empirical Sleepiness Scale. The lower the scores the better the functioning. Range = 0–18; ^5^GAD: Generalized Anxiety Disorder Scale, 7: Sores >= 10 indicate a possible anxiety disorder; ^6^EPDS: The Edinburgh Postnatal Depression Scale: Scores >= 13 indicate possible depression.

**Table 2:** Frequencies and evolution of sleep and daytime variables (*n* = 37).

Severity	ISI		Difficulty initiating or maintaining sleep (DIMS)						Daytime variables					
	2 Months	6 Months	2 Months	6 Months	2 Months	6 Months	2 Months	6 Months	2 Months	6 Months	2 Months	6 Months	2 Months	6 Months
	Insomnia Severity Index		Sleep onset problem^1^		Sleep maintenance problem^1^		Waking too early^1^		Nonrefreshing sleep^1^		Sleepiness^2^		Fatigue^3^	
None	15	20	25	23	23	19	15	22	17	20	19	29	23	26
Mild^4^	16	11	8	10	5	14	10	9	12	11				
Moderate^5^	6	6	4	3	8	2	9	4	6	2	18	8	14	11
Severe^6^	0	0	0	1	1	2	3	2	2	4				

^1^From the Sleep Symptom Checklist (SSC): rating scale: 0–3. ^2^From the ESFS Sleepiness Scale. Moderate/severe refers to scores above the mean (>6.). None/mild severity refers to scores below the mean. ^3^From the ESFS Fatigue Scale. Moderate/severe refers to scores above the mean (>9.). None/mild severity refers to scores below the mean. ^4^Mild severity on the ISI refers to subthreshold insomnia (scores between 8 and 14). ^5^Moderate insomnia on the ISI refers to scores between 15 and 21. ^6^Severe insomnia on the ISI refers to scores between 22 and 28.

**Table 3:** Frequencies of depression and anxiety at 2 and 6 months.

	Depression (EPDS)^1^		Anxiety (GAD)^2^	
	2 months	6 months	2 months	6 months
Below cutoff	33	34	33	34
Above cutoff	4	3	4	3

Frequencies for depression and anxiety are identical; ^1^EPDS: Edinburgh Postnatal Depression Scale: scores >= 13 indicated possible depression; ^2^GAD: Generalized Anxiety Disorder Scale-7: scores >= 10 indicate a possible anxiety disorder.

**Table 4:** Correlations of daytime variables with mothers' sleep characteristics.

	2 months			6 months		
	Fatigue (ESFS)	Sleepiness (ESFS)	Nonrefreshing sleep (SSC)	Fatigue (ESFS)	Sleepiness (ESFS)	Nonrefreshing sleep (SSC)
Nighttime variables						
Insomnia Severity Scale (ISI)	**.610** ^*∗∗*^	**.476** ^*∗∗*^	**.587** ^*∗∗*^	**.419** ^*∗∗*^	**.396** ^*∗*^	**.761** ^*∗∗∗*^
Difficulty initiating or maintaining sleep (DIMS)						
Sleep onset problem (SSC)	.166	.059	.317	.251	**.370** ^*∗*^	**.553** ^*∗∗∗*^
Sleep maintenance problem (SSC)	−.046	.213	.061	.214	** .310** ^+^	**.595** ^*∗∗∗*^
Waking too early (SSC)	.016	.053	−.069	−.077	−.168	**.352** ^*∗*^
Nighttime TST, mother (diary)	.063	.026	.056	−.203	−.149	−**.463**^*∗∗*^
Number of nighttime sleep episodes, mother (diary)	−.099	−.127	−.032	.003	−.247	.267
Daytime variables						
Daytime TST, mother (diary)	−.042	−.202	−.056	−.013	.071	.075
Number of daytime sleep episodes, mother (diary)	−.104	−.164	−.117	.044	.059	.096

*Note.* Significant correlation coefficients are bolded; ^*∗∗∗*^*p* < .001; ^*∗∗*^*p* < .01; ^*∗*^*p* < .05; ^+^*p* < .10.

**Table 5:** Correlations of depression and anxiety with mothers' daytime and sleep characteristics and with infants' sleep characteristics.

	2 months		6 months	
	Depression (EPDS)	Anxiety (GAD-7)	Depression (EPDS)	Anxiety (GAD-7)
Nighttime variables				
Poor sleep quality (SSC)	**.452** ^*∗∗*^	**.449** ^*∗∗*^	.130	**.431** ^*∗∗*^
Insomnia Severity Scale (ISI)	**.642** ^*∗∗∗*^	**.584** ^*∗∗∗*^	**.287** ^+^	**.488** ^*∗∗*^
Difficulty initiating or maintaining sleep (DIMS)				
Sleep onset problem (SSC)	**.338** ^*∗*^	.182	.111	**.374** ^*∗*^
Sleep maintenance problem (SSC)	**.490** ^*∗∗*^	**.398** ^*∗*^	.024	.157
Waking too early (SSC)	.144	.178	.126	−.029
Nighttime TST, mother (diary)	−.176	−.039	−**.354**^*∗*^	−**.315**^+^
Nighttime TST, infant (diary)	−.165	−.053	−.266	−.184
Number of nighttime sleep episodes, mother (diary)	−.068	−.250	−.014	.086
Number of nighttime sleep episodes, infant (diary)	.015	−.183	−.063	−.072
Daytime variables				
Fatigue Scale (EFSS)	**.536** ^*∗∗*^	**.542** ^*∗∗*^	**.417** ^*∗*^	**.506** ^*∗∗∗*^
Sleepiness Scale (EFSS)	**.304** ^+^	**.484** ^*∗∗*^	**.389** ^*∗*^	**.300** ^+^
Nonrefreshing Sleep (SSC)	**.523** ^*∗∗*^	**.450** ^*∗∗*^	.210	**.354** ^*∗*^
Sleep daytime variables				
Daytime TST, mother (diary)	.148	.026	.085	−.144
Daytime TST, infant (diary)	−.126	−.151	−.002	−.022
Number of daytime sleep episodes, mother (diary)	−.033	−.128	.091	−.186
Number of daytime sleep episodes, infant (diary)	.075	.009	−.059	.135

*Note.* Significant correlation coefficients are bolded; ^*∗∗∗*^*p* < .001; ^*∗∗*^*p* < .01; ^*∗*^*p* < .05; ^+^*p* < .10.

**Table 6:** Correlations between mothers' nighttime and daytime variables and infants' sleep variables.

Mother sleep variables	Infant Sleep Variables							
	2 months				6 months			
	Daytime sleep episodes	Nighttime sleep episodes	Daytime TST	Nighttime TST	Daytime sleep episodes	Nighttime sleep episodes	Daytime TST	Nighttime TST
Nighttime variables								
Poor sleep quality (SSC)	−.244	−**.329**^*∗*^	−.227	−0.120	.202	.228	.000	−.151
Insomnia Severity Scale (ISI)	−.043	−.098	−.171	−0.158	.056	.110	−.196	.015
Difficulty initiating or maintaining sleep (DIMS)								
Sleep onset problem (SSC)	.173	.130	.097	−0.264	.063	.069	−.115	.055
Sleep maintenance problem (SSC)	.030	−.094	.023	−0.036	.159	.018	−.100	.222
Waking too early (SSC)	−.114	−.048	.118	0.185	.249	**.289** ^+^	.146	−.197
Nighttime TST, mother (Diary)	.201	−.107	.008	**.673** ^*∗∗∗*^	−.105	−**.318**^+^	.216	.202
Number of nighttime sleep episodes, mother (diary)	**.489** ^*∗∗*^	**.733** ^*∗∗∗*^	−.129	0.057	.267	**.895** ^*∗∗*^	.159	−.162
Daytime variables								
Fatigue Scale (ESFS)	.075	−.099	−.058	0.063	−.094	−.181	−.223	−.230
Sleepiness Scale (EFSS)	.020	−.127	−.130	0.026	−**.285**^+^	−**.308**^+^	−**.361**^*∗*^	−.013
Nonrefreshing sleep (SSC)	−.072	−.032	−.320	0.056	.175	.183	−.231	.080
Sleep daytime variables								
Daytime TST, mother (diary)	−**.308**^+^	−.149	.182	−**.602**^*∗∗*^	−.150	.098	.048	−**.314**^+^
Number of daytime sleep episodes, mother (diary)	−.124	.044	.185	−**.497**^*∗∗*^	−.176	.218	.030	−.262
Mood variables								
Depression (EPDS)	.075	.015	−.126	−0.165	−.059	−.063	−.002	−.266
Anxiety (GAD)	.009	−.183	−.151	−0.053	.135	−.072	−.022	−.184

*Note*. Significant correlation coefficients are bolded; ^*∗∗∗*^*p* < .001; ^*∗∗*^*p* < .01; ^*∗*^*p* < .05; ^+^*p* < .10.
